# Computer-generated holograms for complex surface reliefs on azopolymer films

**DOI:** 10.1038/s41598-019-43256-w

**Published:** 2019-05-01

**Authors:** Stefano Luigi Oscurato, Marcella Salvatore, Fabio Borbone, Pasqualino Maddalena, Antonio Ambrosio

**Affiliations:** 1 Department of Physics “E. Pancini”, University of Naples “Federico II”, Complesso Universitario di Monte Sant’Angelo, Via Cintia, 80126 Naples, Italy; 2 Department of Chemical Sciences, University of Naples “Federico II”, Complesso Universitario di Monte Sant’Angelo, Via Cintia, 80126 Naples, Italy; 3 Center for Nanoscale Systems, Harvard University, 9 Oxford Street, Cambridge, Massachusetts 02138 United States

**Keywords:** Lithography, Surface patterning, Polymers

## Abstract

The light-driven superficial structuration observed on the surface of films of azobenzene-containing polymers follows the optical field distribution of the illuminating light pattern, i.e. the light polarization state and the intensity distribution. The ability to precisely manipulate the illuminating intensity pattern can hence provide a new level in the range of complex light-induced superficial textures accessible onto azopolymer film surfaces. In this respect, digital holography, based on the principles of the Computer-Generated Holograms (CGHs), and actually implemented by means of a versatile liquid crystal spatial light modulator, can represent a unique experimental tool in the field of the light-induced mass migration in azo-materials. In the present work, we demonstrate the possibility to precisely control the features and the quality of complex light patterns generated through CGHs in order to induce arbitrarily complex surface reliefs onto the surface of an azopolymer. The results shown here can potentially broaden the range of possible applications of photo-responsive azopolymer films in the fields of surface engineering, biology and photonics.

## Introduction

Azopolymer films develop superficial reliefs in response to irradiation with UV/visible light. The phenomenon is described in terms of a light-driven macroscopic mass transport of the polymer chains, triggered by the microscopic photo-isomerization dynamics of the azobenzene molecules embedded into the polymeric matrix^1–3^. The mass migration takes place only in illuminated areas of the surface and it is highly directional, with a very peculiar sensitivity to polarization and intensity distributions of the irradiating light field. When the illuminating light is linearly polarized, the material displacement mainly occurs in the light polarization direction. Illumination with circularly polarized light induces instead an isotropic motion of the polymer along the direction of the intensity gradients in the light pattern^1–3^. This polymer motion proceeds from light maxima to light minima, molding in fact the azopolymer surface with the same spatial profile of the illumination pattern.

Since its discovery in 1995^4,5^ with the sinusoidal surface reliefs (*Surface Relief Gratings*, SRGs) induced by the irradiation of azopolymers with the interference pattern of two laser beams, the mass migration and the resulting surface reliefs have been largely exploited as light-induced superficial patterning technique^6,7^. Recent advances in this field are now oriented toward the realization of complex superficial textures, particularly suited for applications in wettability, biology and photonics^3,8–15^, and not achievable with standard photoresists that only respond to the light intensity distribution.

Azopolymer textures with a degree of structural complexity higher than the sinusoidal SRGs are typically accomplished by serial illumination steps, examples are: multiple and sequential film exposure^16^; direct laser writing through laser beams scanned across the film^17–19^; light-induced reconfiguration of a properly pre-textured azopolymer surfaces^20–23^. Besides requiring multi-step approaches, all these methods are limited in terms of versatility and complexity of the achievable surface textures. On the contrary, an approach able to inscribe surface reliefs with almost arbitrary shape would be of great relevance for broadening the range of possible applications of the azopolymers in the field of structural surface engineering. A similar method requires the ability of precisely control the complex spatially-structured light patterns used to irradiate the azopolymer film. To this aim, optical schemes based on *Computer-Generated Holograms* (CGHs)^24^, implemented through liquid-crystal Spatial Light Modulators (LC-SLM), are very promising for a complex and single-step structuration of the azopolymer surfaces^25,26^. In the present work, we demonstrate for the first time the inscriptions of high-quality complex surface reliefs having controlled predictable geometries and dimensions, which are achieved through the proper design of the CGH optical scheme and a speckle noise suppression technique.

## Results and Discussion

### Hologram design and optical setup

**Figure 1:**
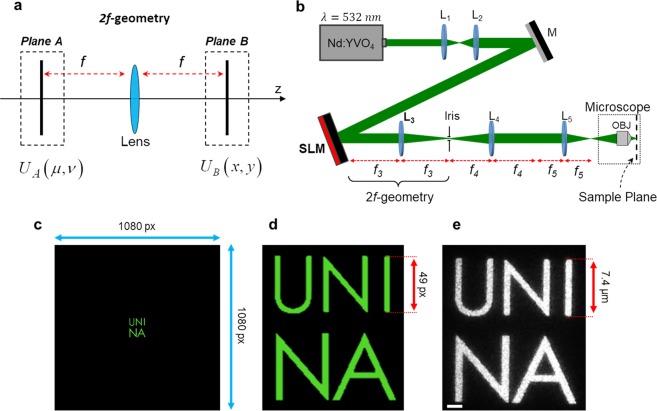
Hologram design and optical setup. (**a**) Schematic illustration of the 2f-geometry for a converging lens used for CGHs. The phase profile *ϕ*_*A*_(*μ*, *ν*) of the optical field *U*_*A*_(*μ*, *ν*) is spatially modulated in the first focal plane (plane A) of the lens in order to reconstruct the desired intensity pattern *I*_*t*_(*x*, *y*) = |*U*_*B*_(*x*, *y*)|^2^ in the second focal plane (plane B). (**b**) Schematic representation of the optical CGH setup used in this work. The Spatial Light Modulator (SLM) for kinoform implementation is placed in the first focal plane of the lens L_3_, which realizes the 2f-geometry with the Iris plane. This plane is conjugated (through the lenses L_4_ and L_5_) to the objective focal plane where the azopolymer film is placed. Segments f_i_ denote the focal lengths of the corresponding lenses L_i_. (**c**) Digital images and (**d**) zoomed view of a target intensity distribution *I*_*t*_(*x*, *y*) designed as 1080 × 1080 px, representing simple text characters. (**e**) Experimental optical image of the light intensity distribution reconstructed in the focal plane of the microscope objective reproducing the pattern of the target image in panels (c,d). Scale bar 2 µm.

Our experimental configuration is based on *phase-only CGHs*, where the reconstruction of a desired holographic intensity pattern in a specific plane of the optical path is achieved by modulating only the wavefront of the light beam. The phase hologram (also named *kinoform*) is accurately calculated using an algorithm based on the Fourier transform relation existing between the complex optical fields in the two focal planes of a converging lens, which is referred to realize a *2f-geometry* (Fig. 1(a))^24,27^. In this configuration, the intensity pattern to be produced in the second focal plane of the lens (plane B in Fig. 1(a)) is specified as a target intensity distribution *I*_*t*_(*x*, *y*), while the phase modulation of the beam occurs in the first focal plane of the lens (plane A in Fig. 1(a)). The hologram calculation in phase-only CGHs requires the calculation of the proper phase profile *ϕ*_*A*_(*μ*, *ν*) (the kinoform) of the beam in the plane A such that the following relation is verified in the plane B:

1 $${|{U}_{B}(x,y)|}^{2}\equiv {I}_{t}(x,y)={(FT\{\exp [i{\varphi }_{A}(\mu ,\nu )]\})}^{2}$$

The phase profile *ϕ*_*A*_(*μ*, *ν*), solution of Eq. (1), is found only in approximated form by iterative algorithms which approximately encode the overall optical field information (amplitude and phase) in a pure phase profile^28^. In the present work we use the Gerchberg-Saxton (G-S) algorithm^29^, which is largely used in situations where the reconstruction of bi-dimensional structured intensity patterns is the goal^28^. One of the advantages of the CGHs in respect to the standard holography is that the target intensity distribution *I*_*t*_(*x*, *y*) entering in Eq. (1) can be built digitally by conventional computer graphics, and can be to such extend arbitrary complex. In typical experimental configurations, the optical setup for CGH uses a phase-only LC-SLM to practically implement the calculated kinoforms.

The schematic representation of the SLM-based setup we used in this work is shown in Fig. 1(b). After a beam expander (lenses L_1_ and L_2_), the laser beam at λ = 532 nm (from Nd:YVO4 continuous-wave frequency-doubled laser) is reflected onto a computer controlled phase-only SLM (Holoeye Pluto) working in reflection mode. The SLM is placed in the first focal plane of the lens L_3_ to realize the 2f-geometry. The beam, diffracted and modulated by the SLM, is then focused in the second focal plane of the lens L_3_, where a first reconstruction of the holographic intensity pattern is obtained. An iris placed in this plane allows the spatial filtering of the beam, rejecting all the undesired light diffraction orders and the un-modulated light emerging from the SLM. After the iris, the beam is re-collimated by the lens L_4_ and finally focused onto the sample located in the microscope (inverted microscope Zeiss Axio-Observer) sample-holder by means of the external lens L_5_ and the microscope objective (OBJ) (oil-immersion 100X, NA = 1.4). When needed, a CCD camera connected to the microscope (not shown in Fig. 1(b)) is able to collect the reflected/back-scattered light in the epi-illumination configuration with the laser beam working as illuminating source^30^. The focal lengths of the lenses L_3_, L_4_ and L_5_ are properly chosen in order to maximize the spatial resolution of the reconstructed intensity pattern. Once those focal lengths are fixed, a specific relation between the dimensions of the optical fields in the SLM and the sample plane exists. This relation has to be used for the design of the target intensity distribution *I*_*t*_(*x*, *y*) in order to build intensity pattern of controlled lateral dimensions in the sample plane, where the azopolymer film will be placed. The relation can be simply derived from the theory of scalar Fourier optics, specified to the case of discrete optical fields (because of the pixelated nature of the LC-SLM used for the hologram implementation).

For optical fields having *M* × *M* discrete sampling points, the Fourier relation deriving from 2f-geometry in paraxial approximation can be written as

2 $${U}_{B}(p{{\rm{\Lambda }}}_{x},q{{\rm{\Lambda }}}_{y})=\alpha \sum _{n=0}^{M-1}\,\sum _{m=0}^{M-1}\,{U}_{A}(n{{\rm{\Delta }}}_{\mu },m{{\rm{\Delta }}}_{\nu })\,\exp \,[-i\frac{k(p{{\rm{\Lambda }}}_{x}n{{\rm{\Delta }}}_{\mu }+q{{\rm{\Lambda }}}_{y}{m{\rm{\Delta }}}_{\nu })}{f}],$$

Where *k* = 2*π*/*λ* is the light wavenumber; *f* is the focal length of the lens in 2f-geometry; *α* is a complex constant containing spatially independent amplitude and phase factors; *p*, *n* = 0 … *M* − 1; *q*, *m* = 0 … *M* − 1 are the pixel indices, Λ_*x*_ and Λ_*y*_ are the dimensions of the pixel in the reconstruction plane along x and y directions and Δ_*μ*_ and Δ_*ν*_ are the dimensions of the pixel in the object plane along *μ* and *ν* directions. Δ_*μ*_ and Δ_*ν*_ are specified by the physical dimensions of the pixels of the light modulating device. As our modulator has square active pixels, we have Δ_*μ*_ = Δ_*ν*_ = Δ. In order to determine the minimum sampling dimension Λ and the overall lateral dimensions *F* = *M*Λ of the field in the reconstruction plane, let’s consider an optical field having lateral dimensions *L* × *L* (with *L* = *M*Δ) in the plane A. Experimentally, such dimensions coincide with the actual illuminated area of the SLM. From relation (2), the field in the reconstruction plane (plane B) has maximum bandwidth^24^ *B* = *L*/*λf*. This quantity defines Λ and *F* in the reconstruction plane through the relations:

3 $${\rm{\Lambda }}=\frac{1}{B}=\frac{\lambda f}{L},$$

4 $$F=M{\rm{\Lambda }}=\frac{\lambda f}{{\rm{\Delta }}}.$$

From relation (3) we can see that Λ depends explicitly on the focal length *f* of the lens as well as on the light wavelength and maximum illuminated area of the SLM. As these two last parameters are typically fixed in the CGH setup, in order to maximize the spatial resolution in the reconstructed optical field, the focal length *f* appearing in Eq. (3) has to be properly chosen in such a way that the sampling dimension Λ is comparable with the diffraction limit of the lens. Once the focal length *f* is chosen, Eq. (4) can then be used then to know the overall lateral dimensions of the holographic pattern and to set a quantitative relation between the pixels in the digital image of the target intensity *I*_*t*_(*x*, *y*) and the physical dimensions of the optical field in the hologram reconstruction plane.

In our experimental configuration, the focal length entering in the Eqs (3) and (4) is the equivalent focal length *f*_*eq*_ = *f*_3_*f*_5_/*f*_4_ of the three lenses L_3_, L_4_ and L_5_ in the telescope configuration shown in Fig. 1(b). As the area of the SLM effectively illuminated is approximately 1080 × 1080 pixels, we have *L* = 8.64 *mm* (where we have considered the physical dimension Δ = 8 *μm* of the pixel of our SLM). Choosing *f*_3_ = 400 *mm*, *f*_4_ = 400 *mm* and *f*_5_ = 250 *mm*, we have *f*_*eq*_ = 250 *mm* and the field in the focal plane of the lens L_5_ is diffraction limited (Λ < *d*, where *d* = *λf*_*eq*_/*D* is the diffraction limit and *D* = 2.54 *cm* is the diameter of the lenses). From Eq. (4), the overall field dimension corresponding to the digital representations of target intensity patterns *I*_*t*_(*x*, *y*) constituted by grayscale images of 1080 × 1080 pixels is *F* = 16.6 *mm* in the focal plane of lens L_5_. A simple proportionality relation can give the physical dimensions of smaller features in the 1080 × 1080 target intensity image.

In order to prove the precise control on the physical dimensions of the reconstructed holographic intensity patterns achieved though this setup design, in Fig. 1(c–e) we compare the experimentally measured dimensions of a holographic intensity pattern constituted by simple text letters of the word “UNINA” and the corresponding dimensions predicted from Eqs (3) and (4). In particular, considering explicitly the character “I”, which has been designed as a 49 pixel long bright pixel area (Fig. 1(d)) in the 1080 × 1080 digital intensity target image (Fig. 1(c)), from Eq. (4) we should have a physical dimension of F^*^ = *F* × 49/1080 = 0.75 *mm* for the corresponding intensity feature in reconstructed optical field in the focal plane of the lens L_5_. By focusing this field through the 100X objective, the dimension in the sample plane is expected to be *F*_*focused*_ = 7.5 *μm*. In these estimations, we have neglected any effect of light diffraction. The calculated dimension has to be compared with the measured length *F*_*meas*_ = 7.4 *μm* from the microscope optical image (Fig. 1(e)) obtained by collecting, through a CCD camera, the holographic light pattern reflected by a silver mirror placed in the focal plane of the microscope objective. The agreement between the predicted and the measured distances is excellent, confirming our ability in designing high-resolution intensity patterns of desired complexity and dimensions.

### Speckle noise reduction procedure.

**Figure 2:**
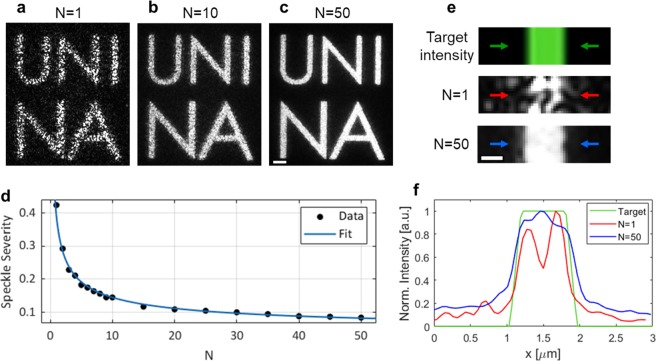
Speckle noise reduction though the time averaging of independent kinoforms. Summed images of the holographic intensity pattern reflected by a silver mirror as obtained for (**a**) N = 1, (**b**) N = 10, (**c**) N = 50 time-averaged independent kinoforms. Scale bar 2 um. (**d**) Plot of the speckle severity S measured in each image as function of N. The blue line represents the fitted curve from Eq. (6). (**e**) Zoomed view of the regions corresponding to the center of the character “I” for the target intensity image (top) and averaged holographic patterns obtained for N = 1 (center) and N = 50 (bottom). Scale bar 500 nm. (**f**) Intensity plot traced along the cross sections indicated by the colored arrows in the three images of panel (e).

The high quality experimental image show in Fig. 1(e) is not the image of a simple reconstructed CGH, but it is the result of a noise reduction procedure which is necessary to effectively use CGHs for the realization of complex surface reliefs on azopolymers. In fact, every CGH reconstructed from Eq. (1) through the G-S algorithm suffers of grainy noise (the *speckle* *noise*) superimposed to the desired intensity pattern (Fig. 2(a)).

The speckle noise^31^ in the reconstructed holograms arises from the random phase profile necessary in the G-S algorithm to guarantee the convergence^32^ of the solution of Eq. (1). However, the random nature of this holographic noise allows us to use a very simple approach for its reduction based on time average of the random speckle patterns. The temporal average can be realized by sequentially displaying several independent kinoforms calculated from the same target intensity profile^30,33–35^ at the maximum refresh rate allowed by the SLM. The result of such procedure is an homogenous averaged holographic intensity pattern in which the granularity of the speckle noise affecting each individual reconstructed pattern is drastically reduced in time^30,34,36^. Such noise-compensating procedure is appropriate for all the applications where the *corrected* holographic light pattern is used to trigger a physical process slower than the possible refresh-rate of the SLM (the maximum refresh-rate of our SLM is 60 Hz). This speckle averaging approach has been already successfully demonstrated for the holographic patterning of graphene-oxide films through light-driven reduction^34^ and can be of great relevance also for the complex surface structuring of azopolymers through the light-driven mass transport phenomenon, which occurs on time scales ranging from seconds to minutes.

The physical parameter commonly used to characterize the quality of images affected by the speckle noise is the dimensionless *speckle severity S*^37,38^ defined as:

5 $$S=\frac{\sigma }{\langle I\rangle }.$$

In Eq. (5), $\langle I\rangle$ is the mean intensity and *σ* is its standard deviation measured in the image. From the definition, it follows that the more the intensity distribution in the image is granular (higher values of σ), higher is the value of the parameter *S*. As the speckle noise reduction method used here involves the average of a number N of statistically independent random variables (the initializing random kinoforms of the G-S algorithm), the expected behavior of the parameter S as function of N is^30,34,36^:

6 $$S(N)={C}_{0}+\frac{{C}_{1}}{\sqrt{N}}.$$

In order to experimentally characterize the reduction of the speckle noise in our CGH setup through the kinoform time-averaging approach, we analyzed the features of the holographic intensity light pattern presented in Fig. 1. In this experiment, the sample is constituted by a silver mirror, placed in the focal plane of the microscope objective, which reflects the holographic illumination pattern. The reflected light is collected by the CCD camera, which is programmed to acquire a stack of fixed number of 200 frames. In each experiment, the number N of independent kinoforms displayed cyclically (with a refresh rate of 5 Hz) onto the SLM is variated from N = 1 to N = 50. For each N, a single image (the *summed image*) is reconstructed by summing together all the 200 frames in the acquired stack. In Fig. 2(a–c) are presented some of the summed images of the holographic intensity patterns obtained for different number N of time-averaged kinoforms. Comparing the images in Fig. 2(a) and in Fig. 2(c), it is clear that the granularity due to speckle noise is visibly reduced passing from N = 1 to N = 10 to N = 50 averaged kinoforms. Also the speckle severity measured in the images is sensibly reduced, showing a monotonically decreasing behavior at increasing values of N which well recovers the expected $1/\sqrt{N}$ trend, as demonstrated by the good agreement between the experimental data and the fit model plotted in Fig. 2(d). The improvement in the quality of the time-averaged holographic pattern resulting from speckle time-averaging can also be highlighted from the comparison of the intensity profiles (Fig. 2(f)) traced across the character “I” of the holographic summed images (Fig. 2(e)). The intensity cross section obtained from N = 50 averaged kinoforms (blue line) has less fluctuations than the holographic pattern obtained from N = 1 (red line), and it is closer to the desired target intensity profile (green line) of the digital target image shown in the top panel of Fig. 2(e).

**Figure 3:**
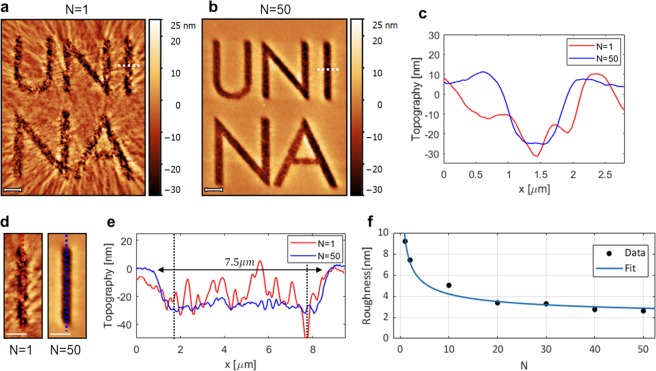
Effects of the speckle noise suppression on the quality of the surface reliefs inscribed on the azopolymer film. AFM images of the reliefs obtained for (**a**) N = 1 and (**b**) N = 50 averaged holographic patterns. Scale bars 2 *μm*. (**c**) Topographic plots traced along the dashed white lines in the AFM images of panel (a) (red line) and (**b**) (blue line). (**d**) zoomed views and (**e**) relative topographic profiles traced along the colored dashed lines of the character “I” in the reliefs obtained for N = 1 (red) and N = 50 (blue). The vertical lines in the plot indicate the regions used for roughness measurement. (**f**) Plot of the measured roughness as function of N showing $1/\sqrt{N}$ behavior.

As the light-induced mass migration of azopolymers strongly depends on the distributions of intensity gradients over the illuminated area of the film, the reduction of holographic speckle noise demonstrated here is crucial for a successful use of CGHs in the realization of high-quality complex surface reliefs. This can be clearly understood from the topographic characterization of the surface reliefs obtained on the surface of a 500 nm thick azopolymer film irradiated with the “UNINA” holographic patterns characterized above. The azopolymer used here has been already fully characterized in previous works^23,25^. In this experiment, different areas of the polymer film have been irradiated for each N, keeping constant both the incident light power (P = 0.45 mW) and the exposure time (t = 100 s). The polarization of the light illuminating the polymer is turned into circular by placing a quarter waveplate in the optical path before the microscope entrance. The resulting surface reliefs have been characterized using an Atomic Force Microscope (AFM) (WITec Alpha RS300^39,40^) operating in tapping mode. Figure 3(a,b) show the AFM images of the surface reliefs obtained for N = 1 and N = 50, respectively. In the case of film irradiation with the N = 1 holographic pattern, the granular intensity due to the not-compensated speckle noise produces a rough surface modulation, which is instead drastically reduced when the film is irradiated with N = 50 consecutive kinoforms. Figure 3(c) shows the topographic cross sections traced along the dashed white lines in Fig. 3(a,b). In the case of irradiation with N = 50 independent holographic patterns, a clear symmetric surface relief of about 30 nm in depth reproducing the character “I” is obtained. On the contrary, the granular intensity noise affecting the pattern with N = 1 does not allow to recognize a definite shape in the final surface relief (Fig. 3(c)).

The improvement in the quality of the surface reliefs can be characterized by analyzing the surface roughness in reference regions of the pattern (e.g. along the character “I”). Figure 3(d) shows the zoomed view of the analyzed surface relief area for N = 1 and N = 50, while Fig. 3(e) reports the relative topographic profiles traced along the vertical dashed lines in the AFM images. The surface roughness is measured as standard deviation of 6 um long topographic profiles traced along the axis of the relief (indicated by the vertical dashed lines in Fig. 3(e)). The roughness measured from the AFM images of the reliefs obtained from the irradiation of holographic patterns with different number N of averaged kinoforms is plotted in Fig. 3(f). This is reduced from 9 nm to 3 nm passing from N = 1 to N = 50 (Fig. 3(f)), while an overall $\sim \,C/\sqrt{N}$ behavior is obtained also in this case, as confirmed by the agreement of the fitted model to the data in Fig. 3(f). Furthermore, the measured length (7.5 *μm*) of the surface relief for N = 50 (Fig. 3(e)) is in perfect agreement with the dimensions expected from the holographic design described above.

### Complex holographic surface reliefs

**Figure 4:**
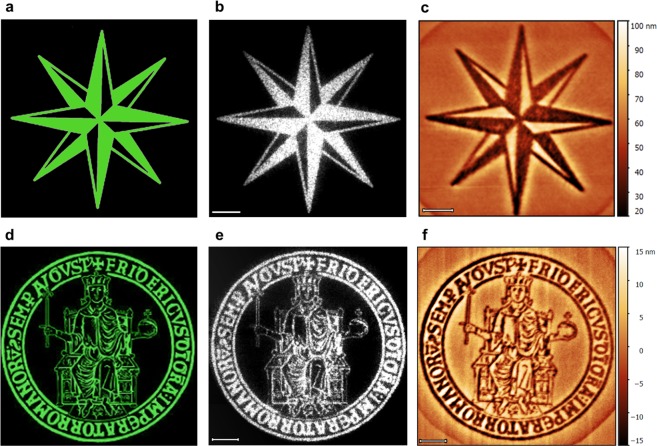
High-quality complex surface reliefs produced by structured holographic light patterns. (**a**) and (**d**) show the digital images of the two complex patterns representing the target intensity distributions *I*_*t*_(*x*, *y*) used for kinoform calculation. (**b**) and (**e**) are the experimental holographic summed optical images of the time-averaged light patterns used for the irradiation of the azopolymer film corresponding to the target intensities in panel (a) and (d), respectively. These images are reconstructed from summation of 200 CCD-collected frames of N = 50 independent holographic light patterns reflected by a mirror placed in the microscope focal plane. (**c**) and (**f**) AFM images of the corresponding complex holographic surface relief inscribed in a single illumination step onto the surface of the azopolymer film. The laser was circularly polarized. The power was 0.7 mW and the exposure time 8 min for both the experiments. Scale bars 5 *μm*.

Once the ability to precisely control the dimensions and the quality of the surface reliefs has been demonstrated, the potential in the use of CGHs for the inscription of more complex surface reliefs in a single illumination step can be proven. Figure 4 shows the design and the experimental realization of two holographic surface reliefs reproducing a compass rose and the logo of our institution, which serve as examples of arbitrary complex surface reliefs achievable with this illumination scheme. Figure 4(a and d) show the images of the digital target intensity patterns *I*_*t*_(*x*, *y*) used as input for the kinoform calculation algorithm and realized by standard computer software for graphics manipulation. The corresponding experimental time-averaged holographic light patterns (obtained for N = 50 independent kinoforms) in the microscope objective plane are shown Fig. 4(b and e). These are the summed images of 200 CCD-recorded frames of the reconstructed light field reflected by a silver mirror placed in the sample plane. The illumination of the azopolymer film with these holographic light patterns creates high-quality reproductions of the target images of Fig. 4(a and d) in form of modulation of surface topography of the azopolymer. The AFM images of the azopolymer surface reliefs obtained from the irradiation of circularly polarized light with laser power of 0.7 mW and exposure time of 8 min are shown in Fig. 4(c and f), respectively. These reliefs have a degree of structural complexity never reached before in the structuration of azopolymer films with other standard illumination schemes. It is worth remaking here that the depth of the surface reliefs can be controlled by the irradiance of the illuminating light and that the surface structuration arising from the light-induced mass migration of the azopolymer is not related to any destructive process at the azopolymer surface, allowing in principle even reversible light-induced surface modulations.

## Conclusions

In the present work we have demonstrated the possibility offered by a CGH scheme in achieving single-step complex and controlled surface reliefs onto an azopolymer film through the light-induced mass migration phenomenon occurring in these materials. The CGHs allow the accurate design of structured intensity patterns with desired lateral dimensions, while a time-averaging speckle noise reduction method allows the transfer of the holographic intensity patterns into surface reliefs with high quality. In the typical illumination configuration, the highly focused illumination pattern (obtained here through a 100X microscope objective) allows to reach sub-micrometer spatial resolutions for the inscribed surface reliefs, opening to possible use of this illumination configuration in photo-lithography. However, scalable surface reliefs can be simply achieved by using microscope objectives with smaller magnification factors. The results shown here can be of relevance for applications of azopolymers in the fields of wettability, adhesion, biology and photonics, where also the reversibility of the non-destructive light-induced mass migration phenomenon could be exploited.

## Data Availability

The datasets generated during and/or analysed during the current study are available from the corresponding author on reasonable request.

## References

[1] Natansohn A, Rochon P (2002). Photoinduced Motions in Azo-Containing Polymers. Chem. Rev..

[2] Yager KG, Barrett CJ (2006). Novel photo-switching using azobenzene functional materials. J. Photochem. Photobiol. Chem..

[3] Oscurato SL, Salvatore M, Maddalena P, Ambrosio A (2018). From nanoscopic to macroscopic photo-driven motion in azobenzene-containing materials. Nanophotonics.

[4] Rochon P, Batalla E, Natansohn A (1995). Optically induced surface gratings on azoaromatic polymer films. Appl. Phys. Lett..

[5] Kim DY, Tripathy SK, Li L, Kumar J (1995). Laser‐induced holographic surface relief gratings on nonlinear optical polymer films. Appl. Phys. Lett..

[6] Yadavalli NS, Saphiannikova M, Lomadze N, Goldenberg LM, Santer S (2013). Structuring of photosensitive material below diffraction limit using far field irradiation. Appl. Phys. A.

[7] Yadavalli NS, Saphiannikova M, Santer S (2014). Photosensitive response of azobenzene containing films towards pure intensity or polarization interference patterns. Appl. Phys. Lett..

[8] Ambrosio A, Girardo S, Camposeo A, Pisignano D, Maddalena P (2013). Controlling spontaneous surface structuring of azobenzene-containing polymers for large-scale nano-lithography of functional substrates. Appl. Phys. Lett..

[9] Galinski H (2014). Instability-induced pattern formation of photoactivated functional polymers. Proc. Natl. Acad. Sci..

[10] Fedele C, De Gregorio M, Netti PA, Cavalli S, Attanasio C (2017). Azopolymer photopatterning for directional control of angiogenesis. Acta Biomater..

[11] Priimagi A, Shevchenko A (2014). Azopolymer-based micro- and nanopatterning for photonic applications. J. Polym. Sci. Part B Polym. Phys..

[12] Lee S, Kang HS, Park J-K (2012). Directional Photofluidization Lithography: Micro/Nanostructural Evolution by Photofluidic Motions of Azobenzene Materials. Adv. Mater..

[13] Nivas JJ (2017). Effects of ambient air pressure on surface structures produced by ultrashort laser pulse irradiation. Opt. Lett..

[14] Avossa J (2019). Forming nanostructured surfaces through Janus colloidal silica particles with nanowrinkles: A new strategy to superhydrophobicity. Appl. Surf. Sci..

[15] Allahyari E (2019). Laser surface texturing of copper and variation of the wetting response with the laser pulse fluence. Appl. Surf. Sci..

[16] Guo M, Xu Z, Wang X (2008). Photofabrication of Two-Dimensional Quasi-Crystal Patterns on UV-Curable Molecular Azo Glass Films. Langmuir.

[17] Ambrosio A (2010). Realization of submicrometer structures by a confocal system on azopolymer films containing photoluminescent chromophores. J. Appl. Phys..

[18] Rianna C (2015). Reversible Holographic Patterns on Azopolymers for Guiding Cell Adhesion and Orientation. ACS Appl. Mater. Interfaces.

[19] Rianna C (2016). Spatio-Temporal Control of Dynamic Topographic Patterns on Azopolymers for Cell Culture Applications. Adv. Funct. Mater..

[20] Lee S, Kang HS, Ambrosio A, Park J-K, Marrucci L (2015). Directional Superficial Photofluidization for Deterministic Shaping of Complex 3D Architectures. ACS Appl. Mater. Interfaces.

[21] Pirani F (2016). Light-Driven Reversible Shaping of Individual Azopolymeric Micro-Pillars. Sci. Rep..

[22] Yadavalli NS (2016). A comparative study of photoinduced deformation in azobenzene containing polymer films. Soft Matter.

[23] Oscurato SL, Borbone F, Maddalena P, Ambrosio A (2017). Light-Driven Wettability Tailoring of Azopolymer Surfaces with Reconfigured Three-Dimensional Posts. ACS Appl. Mater. Interfaces.

[24] Goodman, J. W. *Introduction to Fourier Optics* (Roberts and Company Publishers, 2005).

[25] Ambrosio A, Marrucci L, Borbone F, Roviello A, Maddalena P (2012). Light-induced spiral mass transport in azo-polymer films under vortex-beam illumination. Nat. Commun..

[26] Ambrosio A, Maddalena P, Marrucci L (2013). Molecular model for light-driven spiral mass transport in azopolymer films. Phys. Rev. Lett..

[27] Novotny, L. & Hecht, B. *Principles of Nano-Optics* (Cambridge University Press, 2006).

[28] Leach J (2006). Interactive approach to optical tweezers control. Appl. Opt..

[29] Gerchberg RW, Saxton WO (1972). A practical algorithm for the determination of the phase from image and diffraction plane pictures. Optik.

[30] Oscurato SL (2017). New microscopy technique based on position localization of scattering particles. Opt. Express.

[31] Goodman JW (1976). Some fundamental properties of speckle. J. Opt. Soc. Am..

[32] Leonardo RD, Ianni F, Ruocco G (2007). Computer generation of optimal holograms for optical trap arrays. Opt. Express.

[33] Amako J, Miura H, Sonehara T (1995). Speckle-noise reduction on kinoform reconstruction using a phase-only spatial light modulator. Appl. Opt..

[34] Orabona E (2014). Holographic patterning of graphene-oxide films by light-driven reduction. Opt. Lett..

[35] Salgado-Remacha FJ (2016). Reducing the variability in random-phase initialized Gerchberg-Saxton Algorithm. Opt. Laser Technol..

[36] Locatelli M (2013). Imaging live humans through smoke and flames using far-infrared digital holography. Opt. Express.

[37] Draijer M, Hondebrink E, van Leeuwen T, Steenbergen W (2009). Review of laser speckle contrast techniques for visualizing tissue perfusion. Lasers Med. Sci..

[38] Boas, D. A. & Dunn, A. K. Laser speckle contrast imaging in biomedical optics. *J. Biomed. Opt*. **15** (2010). 10.1117/1.3285504 PMC2816990 20210435

[39] Alfieri ML (2018). Structural Basis of Polydopamine Film Formation: Probing 5,6-Dihydroxyindole-Based Eumelanin Type Units and the Porphyrin Issue. ACS Appl. Mater. Interfaces.

[40] Alfieri ML (2018). The Chemistry of Polydopamine Film Formation: The Amine-Quinone Interplay. Biomimetics.

